# Estimated GFR in autosomal dominant polycystic kidney disease: errors of an unpredictable method

**DOI:** 10.1007/s40620-022-01286-0

**Published:** 2022-03-31

**Authors:** Rosa Miquel Rodríguez, Sergio Luis-Lima, Juan Manuel Fernandez, María Vanesa Pérez Gómez, Beatriz González Toledo, Marian Cobo, Patricia Delgado-Mallén, Beatriz Escamilla, Cristina Oramas Marco, Sara Estupiñán, Coriolano Cruz Perera, Natalia Negrín Mena, Laura Díaz Martín, Sergio Pitti Reyes, Ibrahim Hernández González, Federico González-Rinne, Alejandra González-Delgado, Carmen Ferrer-Moure, Begoña López-Botet Zulueta, Armando Torres, Jose Carlos Rodriguez Pérez, Flavio Gaspari, Alberto Ortiz, Esteban Porrini

**Affiliations:** 1 Nephrology Department, Hospital Universitario de Canarias, Tenerife, Spain; 2 Department of Nephrology and Hypertension, IIS-Fundación Jimenez Díaz, UAM, Madrid, Spain; 3 Nephrology Department, Hospital Universitario Dr Negrín, Las Palmas, Spain; 4 Research Unit, Hospital Universitario de Canarias, Calle Ofra s/n 38023 La Laguna, Tenerife, Spain; 5 Central Laboratory, Hospital Universitario de Canarias, Tenerife, Spain; 6 Internal Medicine Department, Faculty of Medicine, Universidad de La Laguna, Tenerife, Spain; 7 Instituto de Tecnología Biomédicas, ITB, Universidad de La Laguna, La Laguna, Spain; 8 University of Las Palmas de Gran Canaria, Las Palmas de Gran Canaria, Spain; 9 Laboratory of Renal Function, Faculty of Medicine, University of La Laguna, La Laguna, Spain; 10 Red de Investigación Renal (REDINREN), Instituto Carlos III-FEDER, 28040 Madrid, Spain; 11 Radiology Unit, Hospital Universitario de Canarias, Tenerife, Spain

**Keywords:** ADPKD, Chronic kidney disease, Glomerular filtration rate

## Abstract

**Background:**

Autosomal dominant polycystic kidney disease (ADPKD) causes about 10% of cases of end stage renal disease. Disease progression rate is heterogeneous. Tolvaptan is presently the only specific therapeutic option to slow kidney function decline in adults at risk of rapidly progressing ADPKD with chronic kidney disease (CKD) stages 1–4. Thus, a reliable evaluation of kidney function in patients with ADPKD is needed.

**Methods:**

We evaluated the agreement between measured (mGFR) and estimated glomerular filtration rate (eGFR) by 61 formulas based on creatinine and/or cystatin-C (eGFR) in 226 ADPKD patients with diverse GFR values, from predialysis to glomerular hyperfiltration. Also, we evaluated whether incorrect categorization of CKD using eGFR may interfere with the indication and/or reimbursement of Tolvaptan treatment.

**Results:**

No formula showed acceptable agreement with mGFR. Total Deviation Index averaged about 50% for eGFR based on creatinine and/or cystatin-C, indicating that 90% of the estimations of GFR showed bounds of error of 50% when compared with mGFR. In 1 out of 4 cases with mGFR < 30 ml/min, eGFR provided estimations above this threshold. Also, in half of the cases with mGFR between 30 and 40 ml/min, formulas estimated values < 30 ml/min.

**Conclusions:**

The evaluation of renal function with formulas in ADPKD patients is unreliable. Extreme deviation from real renal function is quite frequent. The consequences of this error deserve attention, especially in rapid progressors who may benefit from starting treatment with tolvaptan and in whom specific GFR thresholds are needed for the indication or reimbursement. Whenever possible, mGFR is recommended.

**Graphic abstract:**

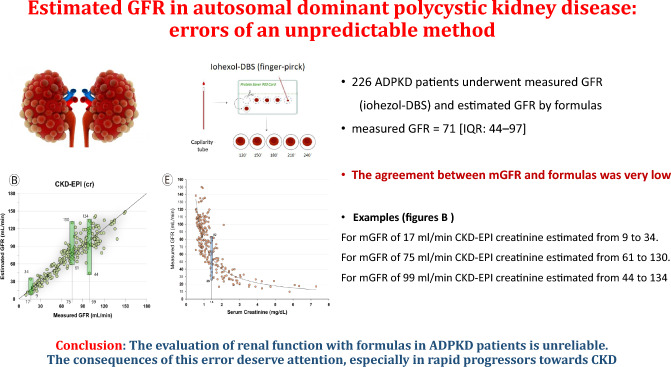

**Supplementary Information:**

The online version contains supplementary material available at 10.1007/s40620-022-01286-0.

## Introduction

Autosomal dominant polycystic kidney disease (ADPKD) is the most frequent inherited nephropathy, causing about 10% of cases with end stage renal disease (ESRD) [1, 2]. The disease is characterized by the formation of renal cysts that progressively increase in size and number replacing normal renal parenchyma [3]. Its pathogenesis is mainly related to changes in the PKD1 and PKD2 genes with an autosomal dominant inheritance [4]. Nevertheless, 10% are spontaneous mutations [5].

Many factors are involved in the progression towards ESRD in patients with ADPKD: male gender, early appearance of urologic events, hypertension and PKD1 truncated mutations, among the most relevant [6]. Importantly, the evolution of renal function in this disease is not uniform: some patients progress rapidly whereas others progress slowly to ESRD [4]. The change in the volume of the renal cysts is a relevant marker of progression, determining an accelerated loss of renal function. Thus, an adequate distinction between patients with rapid progression from those who remain stable is crucial in clinical practice. This makes the evaluation of renal function in this population highly relevant.

Renal function can be assessed either by the indirect estimation of glomerular filtration rate (GFR) using formulas or direct measurement by gold-standard techniques. More than 70 equations have been described, based on creatinine and/or cystatin-c [7]. However, the reliability of theses equations is far from perfect. The average error of any formula is about ± 30% of measured GFR (mGFR) [7]. Accordingly, in a patient with 60 ml/min, estimated GFR (eGFR) may vary from 42 to 78 ml/min [7]. However, limited information is available on the reliability of formulas in patients with ADPKD. An accurate and precise assessment of GFR is important in this population for many reasons: to have a correct evaluation of renal dysfunction, CKD staging and the evolution of renal function over time, i.e. rapid progressors vs stable patients.

In 2017, the use of tolvaptan, a vasopressin antagonist that acts on V2 receptors in renal tubules was approved in Spain to treat patients with ADPKD to slow cyst enlargement and renal function loss [8]. Thus, a proper evaluation of GFR both to indicate the use of tolvaptan and to evaluate the effect of this drug in the evolution of renal function over time is relevant.

In the present study, we evaluated the reliability of eGFR in reflecting real renal function in a large group of patients with ADPKD with diverse levels of GFR, from predialysis to glomerular hyperfiltration.

## Materials and methods

### Patients and design

This is a cross-sectional study including 226 consecutive ADPKD patients attending the outpatient clinics of three Spanish centres: Hospital Universitario de Canarias (HUC-Tenerife), Hospital Universitario Doctor Negrín (HUDN-Gran Canaria) and Hospital Universitario Fundación Jiménez Díaz (FJD-Madrid). All patients underwent the measurement of GFR by the plasma clearance of iohexol (mGFR) using the dried blood spot (DBS) technique. The agreement between eGFR by different equations and mGFR was tested. The European Renal Best Practice (ERBP) guidelines for initiating therapy with tolvaptan were followed as they were adopted by the Spanish drugs agency (AEMPS) to set the indication and reimbursement of tolvaptan in ADPKD [9, 10].

Inclusion criteria were: (a) age > 18 years; (b) ADPKD according to standard criteria [5]; (c) clinical stability: absence of acute kidney injury, active infectious or cardiovascular diseases three months before inclusion. Exclusion criteria were: (a) allergy to iodine; (b) active malignancy; (c) uraemia or imminent dialysis; (d) severe psychiatric disease (e) pregnancy or lactation.

### Procedures

The plasma clearance of iohexol was calculated as described elsewhere [11, 12] and DBS samples were sent to the University of La Laguna (Tenerife, Spain) for analysis. The plasma clearance of iohexol was calculated as described elsewhere [13].

Measurement of creatinine and cystatin-c: creatinine (mg/dL) was measured by IDMS-traceable creatinine (enzymatic assay) in each centre. cystatin-c levels (mg/L) were measured by immunonephelometry using the BN II System (Siemens Healthcare Diagnostics) at the Central Laboratory of the HUC.

Estimated GFR by formulas: renal function was estimated using 61 equations: 38 creatinine-based, 19 cystatin-C-based, and 4 that use both markers. All the algorithms are available at https://lfr.ecihucan.es/apps/documents/egfr_formulas_v2019feb.pdf.

Clinical variables: were collected in an anonymized online-database: age, gender, weight, height, age at diagnosis of ADPKD, family history of the disease, cystic complications, albuminuria, proteinuria, concomitant diseases, i.e. hypertension, dyslipidaemia, diabetes, hyperuricaemia, smoking, medications, measured and eGFR.

Total kidney volume: total kidney volume was analysed either by (a) multiplanar (axial, coronal, and sagittal) MRI, applying T1 and T2 weighted fast spin echo sequence; with the ellipsoid method recommended by the Mayo Clinic Group [14, 15] in largest diameters length, width, depth of each kidney, including exophytic cysts (equation: total kidney volume{TKV} = π/6 × L × W × D) and/or (b) ultrasound using the formula for volume calculation based on the ellipsoid equation referred (TKV = π/6 × L × W × D) [X] in each centre.

Adjustment by body surface area: we worked with unadjusted GFR alone since adjusting GFR by BSA is prone to errors [16]. When equations gave adjusted GFR, we reversed the adjustment of the result by applying the following formula: GFR unadjusted = GFR adjusted x (BSA/1.73m^2^). BSA was calculated by the Du-Bois and Du-Bois formula.

### Statistical analysis

The performance of formulas was assessed by statistics of agreement for continuous data: concordance correlation coefficient (CCC), total deviation index (TDI) and coverage probability (cp). CCC varies from 0 to 1 and combines components of accuracy and precision [17]. A CCC > 0.90, reflects optimal concordance between measurements. TDI captures a large proportion of data within a boundary for allowed differences between two measurements. Empirical TDI was calculated for a theoretical TDI of 10% and a coverage probability of 90%. According with on the basis of this level of TDI, we defined a priori that the acceptable bias between estimated and mGFR should be at least 10%. This is based on the previous reports and on the reproducibility of the method in our laboratory which is < 7%. Coverage probability varies from 0 to 1 and estimates whether a given TDI is less than a pre-specified fixed percentage.

For statistical analyses we used the statistical package AGP (Agreement Program) v.1.0 (Geiko, SP) available at: https://lfr.ecihucan.es/apps/?dir=agreement_installer. AGP is based on the R code originally developed by Lawrence Lin and YuYue. AGP was developed to simplify the use of the tool given in the R agreement package.

### Sensitivity analyses

To evaluate the impact of the error of eGFR in clinical practice, we analysed two groups of patients: those with mGFR < 30 ml/min -the cut-off point to avoid the use of Tolvaptan according to current guidelines [9, 10] in whom formulas estimated GFR above this threshold, and those with mGFR > 30 ml/min in whom formulas provided values below 30 ml/min. We tested the possibility that eGFR incorrectly allows the treatment with Tolvaptan in subjects with real reduced renal function (< 30 ml/min) as well as the number of cases in whom formulas incorrectly contraindicated the use of Tolvaptan in patients with acceptable GFR (> 30 ml/min).

Finally, to facilitate the understanding of the agreement analysis we evaluated the percentage of cases with specific cut-off of errors between eGFR and mGFR, i.e. < 10%, 10–20%, 20–30% and > 30%.

## Results

### Patients

We included 234 patients: about half were males, the average age was 45 ± 14 years, almost 70% of the subjects had hypertension, 26% dyslipidaemia and 35% were current or former smokers (Online Resource 1). Measured GFR averaged 71 [IQR: 44–97] ml/min in the whole group, 75 (32%) of the cases had mGFR > 90 ml/min, 70 (30%) had values between 60 and 90 ml/min, 55 (24%) between 30 and 60 ml/min and 34 (14%) below 30 ml/min. Mean eGFR according to the most used equations ranged from 66 to 75 ml/min for the MDRD and the CKD-EPI group of formulas. The assessment of TKV concomitant to the measurement of GFR was available in 159 cases (68%) and averaged 1008 ml (IQR: 635–1941) and 1639 (IQR: 866–2742) by high performance ultrasound and MRI, respectively.

### Agreement between measured and estimated GFR

Creatinine-based formulas: TDI averaged 55% for all formulas (Table 1). For example, MDRD and CKD-EPI formulas had a TDI of 42% and 39%, respectively, indicating that 90% of the estimations showed an error ranging from about − 40 to + 40% of mGFR. CCC averaged 0.89, reflecting moderate precision and accuracy. Finally, cp averaged 28 indicating that 72% of the estimations had an error >  ± 10%.

**Table 1:** Agreement analysis between measured (iohexol) and eGFR by 61 formulas

Creatinine-based formulas							
	CCC	TDI	*Cp*		CCC	TDI	*Cp*
Effersøe	0.92 (0.90)	44 (49)	33 (30)	aMDRD	0.93 (0.92)	42 (46)	34 (32)
Edward-White	0.89 (0.86)	51 (56)	29 (27)	Wright	0.91 (0.89)	49 (53)	28 (25)
Jelliffe-1	0.88 (0.85)	63 (70)	25 (23)	MCQ	0.90 (0.88)	57 (63)	26 (24)
Mawer	0.91 (0.89)	52 (56)	27 (25)	Sobh	0.81 (0.78)	85 (92)	14 (12)
Jelliffe-2	0.94 (0.93)	37 (40)	38 (35)	Virga	0.93 (0.92)	39 (43)	36 (33)
Cockcroft-Gault	0.92 (0.90)	46 (51)	30 (28)	CHUQ	0.80 (0.76)	95 (106)	18 (17)
Björnsson	0.89 (0.87)	55 (60)	25 (22)	CKD-EPI-cr	0.94 (0.93)	39 (42)	37 (34)
Mogensen	0.75 (0.70)	121 (135)	15 (14)	Lund-Malmö (LBM)	0.89 (0.87)	57 (62)	26 (24)
Hull	0.91 (0.89)	53 (57)	26 (24)	Lund-Malmö	0.92 (0.90)	46 (50)	30 (28)
Gates	0.94 (0.93)	38 (42)	37 (35)	Lund-1	0.93 (0.91)	38 (42)	37 (34)
Walser	0.93 (0.92)	39 (43)	36 (34)	Lund-2 (LBM)	0.86 (0.83)	69 (75)	20 (18)
Davis Chandler	0.90 (0.88)	53 (59)	28 (26)	Lund-Malmö (Rv)	0.94 (0.93)	37 (40)	38 (35)
Baracskay	0.82 (0.79)	61 (67)	26 (24)	Lund-Malmö (RvLBM)	0.92 (0.90)	47 (52)	30 (28)
Martin	0.90 (0.87)	52 (56)	27 (25)	FAS-cr	0.90 (0.88)	49 (53)	30 (27)

Cystatin-C-based formulas							
	CCC	TDI	*Cp*		CCC	TDI	*Cp*
Le Bricon	0.83 (0.79)	69 (72)	23 (21)	Jonsson	0.89 (0.87)	61 (67)	27 (42)
Tan	0.90 (0.87)	51 (56)	29 (27)	Stevens-1	0.9 (0.89)	49 (54)	30 (28)
Hoek	0.90 (0.88)	48 (53)	31 (29)	Stevens-2	0.92 (0.90)	45 (50)	32 (30)
Larsson	0.91 (0.88)	51 (56)	29 (27)	Tidman	0.89 (0.87)	58 (63)	26 (24)
Perkins	0.72 (0.68)	104 (113)	10 (8)	Grubb-2009	0.81 (0.77)	108 (12)	17 (15)
Orebro	0.82 (0.79)	88 (96)	17 (15)	Hojs	0.84 (0.81)	71 (78)	20 (18)
Grubb-2005	0.86 (0.83)	84 (93)	20 (19)	Grubb-2014 (CAPA)	0.92 (0.90)	47 (52)	31 (29)
Rule-cy	0.90 (0.87)	56 (62)	27 (25)	CKD-EPI-cy	0.93 (0.90)	43 (47)	33 (31)
MacIsaac	0.88 (0.85)	57 (62)	26 (24)	FAS-cy	0.83 (0.80)	70 (77)	21 (19)
Arnal-Dade	0.91 (0.88)	53 (58)	29 (26)				

Creatinine- and cystatin-C-based formulas							
	CCC	TDI	*Cp*		CCC	TDI	*Cp*
Ma	0.93 (0.92)	43 (47)	33 (30)	CKD-EPI-cr-cy	0.95 (0.94)	34 (37)	40 (37)
Stevens	0.95 (0.94)	33 (37)	41(38)	FAS-cr-cy	0.89 (0.87)	51 (56)	27 (24)

*TDI* total deviation index; *CCC* concordance correlation coefficient; *cp* coverage probability

Cystatin-C-based formulas: TDI averaged 64% (Table 1). As an example, the Rule-cy formula had a TDI of 56, meaning that 90% of the estimations had an error ranging from − 56 to + 56% of mGFR. CCC averaged 0.87, reflecting a moderate level of precision and accuracy. Finally, cp averaged 25 indicating that more than 75% of the estimations had an error >  ± 10%.

Creatinine and cystatin-C-based formulas: TDI averaged 40%, CCC 0.93 and cp35 (Table 1).

### Low concordance between mGFR and eGFR

**Fig. 1:**
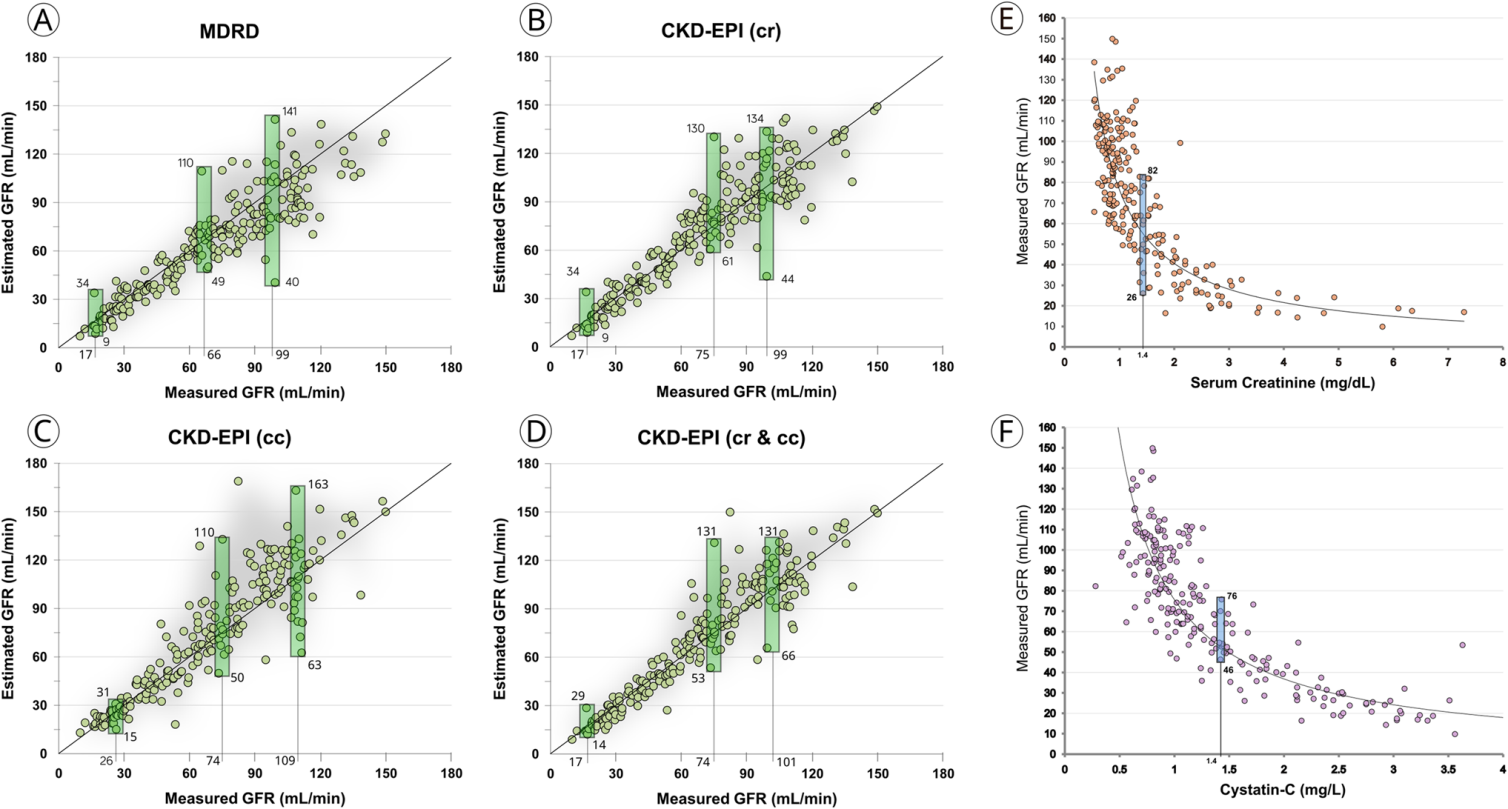
Plot between measured GFR and estimated GFR by four equations MDRD (**A**) and CKD-EPI based on creatinine (**B**), cystatin-c (**C**) or creatinine and cystatin-c (**D**); and serum creatinine (**E**) or cystatin-c (**F**)

Single values of mGFR were associated with an ample range of estimations (Fig. 1). For example, in subjects with 17 ml/min of mGFR, MDRD estimated GFR from 9 to 34 ml/min (Fig. 1A). In patients with moderate CKD, i.e. 75 ml/min, the CKD-EPI formula (creatinine-based) estimated renal function from 61 to 130 ml/min (Fig. 1B). Similar results were observed for the CKD-EPI-cy, CKD-EPI-cr-cy equations (Fig. 1C, D).

### Low concordance between creatinine and cystatin-c with mGFR

The correlation between creatinine or cystatin-c and mGFR was poor (Fig. 1). A single value of creatinine or cystatin-c, i.e. 1.4 mg/dL or mg/l, was associated with a wide range of mGFR: from 26 to 82 and 46 to 76 ml/min, respectively (Fig. 1E and F).

### Evidence of a random error

(a) Examples of eGFR in cases with comparable mGFR: patients were selected in pairs with comparable mGFR covering values from 16 to 95 ml/min (Table 2). The same equation overestimated, underestimated or provided precise estimations for comparable measurement of GFR (Table 2). For cases 1 and 2, who had a similar value of mGFR ~ 16 ml/min, the MDRD and the 3 CKD-EPI formulas overestimated mGFR for case 1 whereas they provided a slight underestimation or accurate values for case 2. In other cases, i.e. 7 and 8 (mGFR ~ 45 ml/min), the same formula underestimated and overestimated mGFR: MDRD: 33 vs 56 ml/min, CKD-EPI-cr: 35 vs 59 ml/min and CKD-EPI-cr-cy: 38 vs 57 ml/min.

**Table 2:** Estimated glomerular filtration rate (GFR) by four equations in 14 ADPKD patients grouped in pairs of similar measured GFR

Case	mGFR	aMDRD	CKD-EPI-cr	CKD-EPI-cy	CKD-EPI-cr-cy
1	16	34	34	25	29
2	17	13	14	17	14
3	20	12	12	20	15
4	21	24	25	22	23
5	29	19	21	23	21
6	30	38	39	28	32
7	45	33	35	43	38
8	46	56	59	56	57
9	66	109	95	93	97
10	68	49	53	62	57
11	78	60	68	65	65
12	80	116	122	107	114
13	95	55	62	58	58
14	95	115	106	98	103

*aMDRD* abbreviated modification of diet in renal disease, *CKD-EPI* chronic kidney disease epidemiology collaboration

**Fig. 2:**
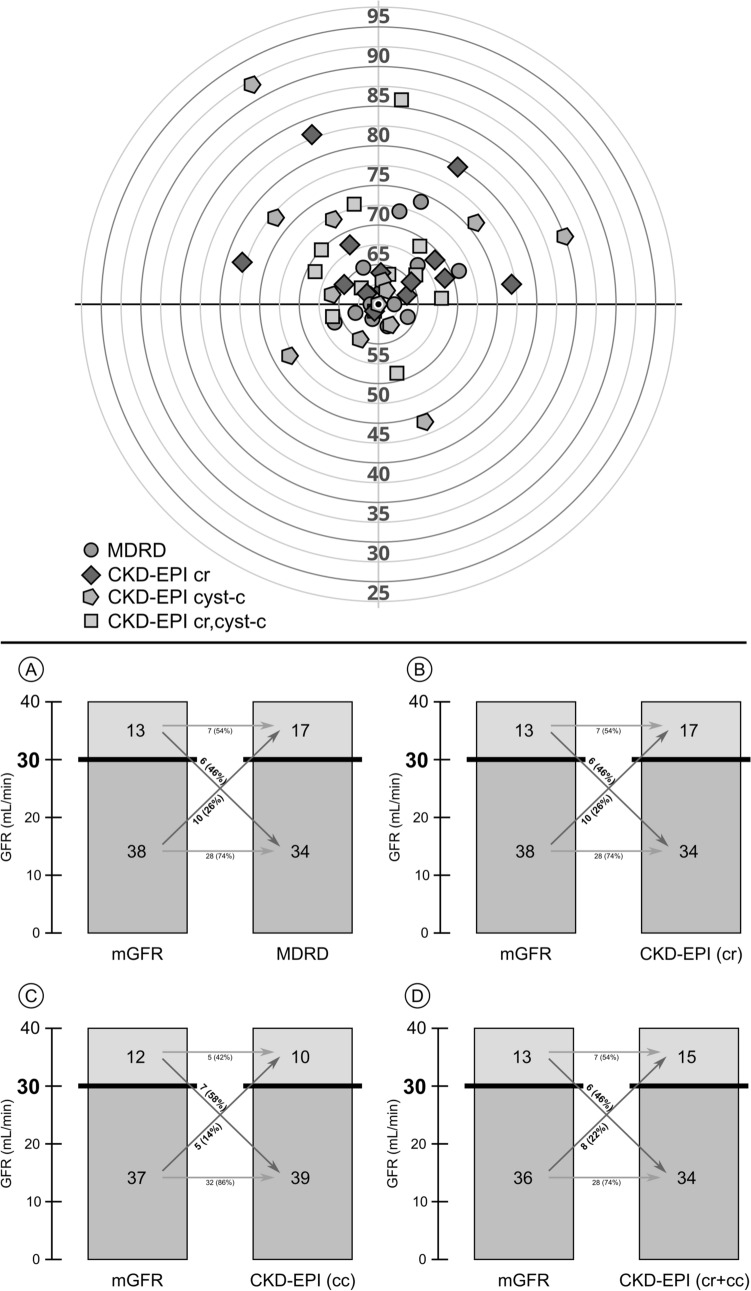
Estimated GFR by four equations in patients with measured GFR of 60 ml/min (N 14)—upper panel-. Estimated GFR by four equations in patients with measured GFR below 40 ml/min. MDRD (**A**) and CKD-EPI based on creatinine (**B**), cystatin-c (**C**) or creatinine and cystatin-c (**D**). Number and percentage of cases in which estimated GFR provided a value of GFR above or below the cut-off of 30 ml/min—lower panel-

(b) Random variations of eGFR around a similar value of mGFR: Fig. 2 (upper panel) illustrates the cases with 60 ml/min of mGFR (*n* = 14) and the estimations of 4 equations: MDRD and the 3 CKD-EPI equations. Estimations were quite scattered, showing either over- or underestimations of real GFR. Extreme variations were frequent with values around 50 and 70 ml/min.

### Sensitivity analysis

A total of 38 cases had mGFR < 30 ml/min. In 10 (26%), the MDRD or the CKD-EPI-creatinine equations provided estimations above this threshold (Fig. 2, lower panels A, B). Furthermore, 13 subjects had mGFR values between 30 and 40 ml/min and in 46% of them, eGFR provided values below 30 ml/min (Fig. 2 lower A, B). Similar results were observed for the CKD-EPI-cystatin, CKD-EPI-cr-cy (Fig. 2 lower panels C, D).

Between 20 and 40% of the estimations with several formulas had an error < 10% of mGFR; 30% of the estimations had an error larger than 10% but < 20% and 30% had an error larger than 20% of mGFR (Online Resource 2).

## Discusion

The main finding of our study was that the error of eGFR formulas in patients with ADPKD was wide, frequent and unpredictable. The average error of any formula was about ± 50% of real renal function. This wide variability was found for every tested equation: either based on creatinine and/or on cystatin-c.

We evaluated a homogeneous group of patients with ADPKD, covering a broad range of renal function, from predialysis care to moderately decreased kidney function to glomerular hyperfiltration. So, the population studied reflects the patients that are seen in day-to-day clinical practice. We evaluated a large group of equations (*n* = 61), in particular those that have been used frequently in the last decades, i.e. MDRD, CKD-EPI, FAS, etc. Thus, we evaluated differences in precision and accuracy between formulas that use creatinine or cystatin-c and the added value of cystatin, if any, in the estimation of GFR. Renal function was measured with a gold standard, the plasma clearance of iohexol. Finally, the agreement between eGFR and mGFR was evaluated with specific statistical tests [17], which supports the reliability of our results.

The main finding of this analysis was that eGFR values were unreliable. For the most frequently used formulas, i.e. MDRD, CKD-EPI-cr, CKD-EPI-cy or CKD-EPI-cr-cy, TDIs were 42%, 38%, 43%, 34%, respectively. Thus, for a patient with mGFR of 60 ml/min, eGFR could vary from 35 to 40 ml/min (− 40%) to 80–85 ml/min (+ 40%). Similar results were observed for the remaining equations. Moreover, in one out of every 10 patients, the error could be even larger. Therefore, formulas have wide and frequent variability in reflecting real renal function in this population. This error is comparable or in some cases larger than the error previously observed in other CKD groups like diabetes, CKD of different origins, renal transplant patients, etc. (see reference 7 for review) [7]. Also, no major differences were found when comparing creatinine-based formulas with each other, i.e. MDRD vs CKD-EPI, which indicates no improvement in the estimation of renal function with modern formulas. Moreover, cystatin-c-based formulas did not offer a real benefit in the evaluation of GFR since the TDI, CCC and cp values were comparable to those equations using creatinine.

The causes of this error are not completely clear. Formulas depend mostly on the relationship between serum creatinine or cystatin-c values and mGFR. However, both markers proved to have a weak correlation with mGFR. A recent analysis showed that a single value of serum creatinine or cystatin-c, i.e. 1.5 mg/dL or mg/L can be associated with a value of mGFR ranging from 30 to 90 ml/min, showing a 200% variability [7]. The same variability has been observed for the correlation between eGFR and mGFR [7] (Fig. 1), meaning that current equations do not correct the error of serum markers. The explanation of this phenomenon is complex. Renal tubular secretion of creatinine increases in CKD leading to GFR overestimation [7]. Also, some authors showed tubular reabsorption of creatinine [7]. Finally, the levels of creatinine are influenced by meat intake, muscle mass and extra renal clearance. Cystatin-c is known to be influenced by inflammation, obesity, hyperthyroidism, older age, and smoking, among others [18–23]. So, many factors may influence serum creatinine or cystatin-c levels independently of the level of renal function. It seems highly unlikely that any mathematical procedure can solve the large error of these markers in reflecting GFR. In conclusion, the error of these markers, the variables with highest weight in the mathematical algorithms, translates into the equations that estimate renal function.

The consequences of the variability of eGFR in patients with ADPKD are multiple. Proper evaluation of the degree of renal dysfunction is crucial in the clinics. The large variability of eGFR may determine that patients can be incorrectly classified in higher or lower CKD stages, as shown previously by our group [24]. Tolvaptan is the only approved specific therapy for patients with ADPKD. Patients who would benefit from tolvaptan treatment are those under 60 years of age who have rapid progression towards advanced CKD (rapid progressors), based on the level of renal function and the value of total kidney volume. According to the European Medicines Agency (EMA), tolvaptan is currently indicated in adults with ADPKD and CKD stage 1–4 (i.e. up to eGFR 15 ml/min) at initiation of treatment with evidence of rapidly progressing disease [25]. However, it also emphasized that limited safety and efficacy data are available in patients with CKD late stage 4 (eGFR < 25 mL/min/1.73 m^2^). As a consequence, some payers still limit reimbursement of the drug based on eGFR values. AEMPS still lists an eGFR value above 45 ml/min/1.73m^2^ to support reimbursement for tolvaptan for ADPKD [9], following outdated ERBP recommendations [10]. More recent Spanish guidelines, not yet adopted by the Spanish Government, state that eGFR > 30 ml/min is a general cut-off point to start the evaluation as a possible candidate to initiate tolvaptan [26]. When eGFR is lower, treatment must be individualized. Finally, based on available placebo-controlled trials, tolvaptan treatment is not recommended in subjects aged from 55 to 60 years and GFR > 60 ml/min [10]. Given the high cost of tolvaptan, these eGFR cut-offs are strictly applied by payers, thus potentially precluding treatment initiation. According to our results, the error of eGFR may have a major impact on the evaluation of candidates to be treated. In subjects with mGFR < 30 ml/min, eGFR could estimate GFR values > 30 ml/min ml/min, or in subjects with 40 ml/min, eGFR may give GFR values < 30 ml/min (Fig. 2). In both cases, and based on our data, it may not be infrequent that a patient can be excluded from treatment or incorrectly treated with very low renal function. The consequences of the error of eGFR in specific interventions in patients with ADPKD deserve more attention in future studies.

Few studies evaluated the error of formulas in ADPKD. Orskov et al. in 101 patients found large p30 values, i.e. the number of estimations included in a range of ± 30% of mGFR, for commonly used formulas: Cockroft-Gault, MDRD and CKD-EPI-cr [27]. A p30 of 80–90%, like that observed in this study, indicates that most of the estimations have a ± 30% variability in reflecting mGFR. Similar results were found by Spithoven et al. in 121 subjects [28]. Finally, Ruggenenti et al. observed large limits of agreement between eGFR (MDRD or CKD-EPI-cr) and mGFR (plasma clearance of iohexol), i.e. from − 30 to 20 ml/min in 111 patients with this disease [29]. So, our study is in line with previous publications showing the poor performance of eGFR in patients with ADPKD.

Our study has limitations. It is a cross sectional study, which does not allow the analysis of the error of eGFR on the evaluation of real GFR changes over time. A prospective evaluation of this cohort will be the matter of a future study.

In conclusion, the evaluation of renal function with formulas in patients with ADPKD is unreliable. Extreme variations from real renal function are quite frequent. The consequences of this error deserve attention as they may preclude reimbursement or initiation of specific therapy for ADPKD. Whenever possible, in particular, to help identify rapid progressors towards CKD or to check the evolution of renal function in patients on Tolvaptan, mGFR is recommended. More research in the field of the consequences of the error of eGFR in this population is clearly needed.

## Supplementary Information

Below is the link to the electronic supplementary material.

Supplementary file1 (DOCX 26 KB)
