# Supplementary material for: Estimated GFR in autosomal dominant polycystic kidney disease: errors of an unpredictable method
Source: J Nephrol. 2022 Mar 31;35(8):2109–18. doi: 10.1007/s40620-022-01286-0 (PMC9584992; doi:10.1007/s40620-022-01286-0)
Supplement: Supplementary file 1 — Supplementary file1 (DOCX 26 KB) [file 40620_2022_1286_MOESM1_ESM.docx]

**Supplementary Information (SI)**

Article title: Estimated GFR in Autosomal Dominant Polycystic Kidney Disease: An unpredictable procedure

Journal name: Journal of Nephrology

Authors: Rosa Miquel Rodríguez ^1^*; Sergio Luis-Lima ^2^*; Juan Manuel Fernandez ^3^*; María Vanesa Pérez Gómez ^2^; Beatriz González Toledo ^2^, Marian Cobo ^1^; Patricia Delgado-Mallén ^1^; Beatriz Escamilla ^1^; Cristina Oramas Marco ^9^; Sara Estupiñán ^1^; Coriolano Cruz Perera ^4^; Natalia Negrín Mena ^4^; Laura Díaz Martín ^9,10^; Sergio Pitti Reyes^11^, Ibrahim Hernández González^11^, Federico González-Rinne ^4^; Alejandra González-Delgado ^5^; Carmen Ferrer-Moure ^5^; Begoña López-Botet Zulueta^2^; Armando Torres ^1,6,7^; Jose Carlos Rodriguez Pérez ^3,8^; Flavio Gaspari ^9^; Alberto Ortiz ^2,10^; Esteban Porrini ^4,6,7,9^

Affiliations:

1. Nephrology Department, Hospital Universitario de Canarias, Tenerife, Spain
2. Department of Nephrology and Hypertension, IIS-Fundación Jimenez Díaz, UAM, Madrid, Spain.
3. Nephrology Department, Hospital Universitario Dr Negrín, Las Palmas de Gran Canaria, Spain
4. Research Unit, Hospital Universitario de Canarias, Tenerife, Spain,
5. Central Laboratory, Hospital Universitario de Canarias, Tenerife, Spain
6. Internal Medicine Department, Faculty of Medicine, Universidad de La Laguna, Tenerife, Spain
7. Instituto de Tecnología Biomédicas, ITB, Universidad de La Laguna
8. University of Las Palmas de Gran Canaria
9. Laboratory of Renal Function, Faculty of Medicine, University of La Laguna
10. Red de Investigación Renal (REDINREN), Instituto Carlos III-FEDER, 28040 Madrid, Spain
11. Radiology Unit, Hospital Universitario de Canarias, Tenerife, Spain

* The first three authors contributed equally to this work.

Corresponding author:

Dr. Esteban Porrini

[esteban.l.porrini@gmail.com](mailto:esteban.l.porrini@gmail.com)

Online Resource 1: Clinical characteristics of the patients included in the study.

| **BASELINE** | | |  |
| --- | --- | --- | --- |
|  | **N** |  | 234 |
|  | **Centre 1 (HUC)** | | 120 (51) |
|  | **Centre 2 (FJD)** | | 65 (28) |
|  | **Centre 3 (HN)** | | 49 (21) |
|  | Age (y) | | 45 (14) |
|  | Sex (N-% males) | | 109 (46) |
|  | Weight (kg) | | 76 (16) |
|  | Height (cm) | | 170 (10) |
|  | Body Mass Index (Kg/m2) | | 26 (5) |
|  |  | Body Mass Index > 30kg/m2 | 47 (20) |
| **Concomitant diseases** | | |  |
|  | Hypertension (N-%) | | 158 (67) |
|  |  | Diuretics | 42 (18) |
|  |  | Beta blockers | 29 (12) |
|  |  | Calcium Antagonist | 27 (11) |
|  |  | ACE inhibitors | 40 (17) |
|  |  | AR blockers | 100 (43) |
|  |  | Symphatholyticagents | 12 (5) |
|  | Dyslipidaemia (N-%) | | 60 (26) |
|  |  | Fibrates | 5 (8) |
|  |  | Statins | 43 (18) |
|  | Diabetes | | 18 (2) |
|  | Hyperuricaemia (N-%) | | 50 (21) |
|  | Smoking* | |  |
|  |  | Never | 145 (62) |
|  |  | Former | 44 (19) |
|  |  | Current | 38 (16) |
| **Kidney function** | | |  |
|  | Measured GFR (ml/min) | | 71 [44-97] |
|  |  | > 90 ml/min (n - %) | 75 (32) |
|  |  | 60-90 ml/min | 70 (30) |
|  |  | 30-60 ml/min | 55 (24) |
|  |  | <30 ml/min | 34 (14) |
|  | Serum creatinine (mg/dL) | | 1.12 [0.9-1.7] |
|  | Serum cystatin-c (mg/L) | | 1.12 [0.8-1.7] |
|  | Proteinuria - mg/24h | | 160 (99-260) |
|  | Albumin/creatinine - mg/g | | 30 (12-99) |
|  | MDRD (ml/min) | | 66 (31) |
|  | CKD-EPI creatinine (ml/min) | | 73 (35) |
|  | CKD-EPI cystatin-c (ml/min) | | 75 (38) |
|  | CKD-EPI creatinine + cystatin-c (ml/min) | | 73 (36) |
| **ADPKD** | | |  |
|  | Age at diagnosis | | 31 [22-44] |
|  | Family history (yes) | | 194 (83) |
|  | Kidney Volume echo | | 1008 (635-1941) |
|  | Kidney Volume MRI** | | 1639 (866-2742) |

ACE: Angiotensin Converting Enzyme; AR: Angiotensin receptors; GFR: glomerular filtration rate; eGFR: estimated glomerular filtration rate; ADPKD: autosomal dominant polycystic kidney disease.* missing data in 7 cases; ** available in 61 patients at the

time of mGFR.

Online Resource 2: Percentage of cases included in different error ranges of eGFR.

|  | **CREATININE-BASED FORMULAS** | | | | |
| --- | --- | --- | --- | --- | --- |
| **bias** | **Effersøe** | **Cockcroft-Gault** | **aMDRD** | **MCQ** | **CKD-EPI-cr** |
| <10% | 35 | 33 | 35 | 19 | 41 |
| 10-20% | 29 | 27 | 37 | 24 | 31 |
| 20-30% | 27 | 15 | 20 | 23 | 17 |
| >30% | 10 | 25 | 8 | 34 | 11 |
|  | **CREATININE- and/or CYSTATIN-C-BASED FORMULAS** | | | | |
| **bias** | **Lund-Malmö (Rv)** | **Rule-cy** | **CKD-EPI-cy** | **CKD-EPI-cr-cy** | **FAS-cr-cy** |
| <10% | 42 | 38 | 39 | 48 | 26 |
| 10-20% | 35 | 28 | 29 | 34 | 30 |
| 20-30% | 15 | 18 | 17 | 12 | 16 |
| >30% | 7 | 16 | 15 | 6 | 28 |
